# A Novel Dimer-Tetramer Transition Captured by the Crystal Structure of the HIV-1 Nef

**DOI:** 10.1371/journal.pone.0026629

**Published:** 2011-11-02

**Authors:** Pankaj Singh, Gaya Prasad Yadav, Sudeepti Gupta, Anil Kumar Tripathi, Ravishankar Ramachandran, Raj Kamal Tripathi

**Affiliations:** 1 Toxicology Division, Central Drug Research Institute (Council of Scientific & Industrial Research), Chattar Manzil, Mahatma Gandhi Marg, Lucknow, Uttar Pradesh, India; 2 Molecular and Structural Biology Division, Central Drug Research Institute (Council of Scientific & Industrial Research), Chattar Manzil, Mahatma Gandhi Marg, Lucknow, Uttar Pradesh, India; 3 Chatrapati Shahuji Maharaj Medical University, Chowk, Lucknow, Uttar Pradesh, India; University of Canterbury, New Zealand of America

## Abstract

HIV-1 Nef modulates disease progression through interactions with over 30 host proteins. Individual chains fold into membrane-interacting N-terminal and C-terminal core (Nef_core_) domains respectively. Nef exists as small oligomers near membranes and associates into higher oligomers such as tetramers or hexadecamers in the cytoplasm. Earlier structures of the Nef_core_ in *apo* and complexed forms with the Fyn-kinase SH3 domain revealed dimeric association details and the role of the conserved PXXP recognition motif (residues 72–78) of Nef in SH3-domain interactions. The crystal structure of the tetrameric Nef reported here corresponds to the elusive cytoplasmic stage. Comparative analyses show that subunits of Nef_core_ dimers (*open* conformation) swing out with a relative displacement of ∼22 Å and rotation of ∼174° to form the ‘*closed*’ tetrameric structure. The changes to the association are around Asp125, a conserved residue important for viral replication and the important XR motif (residues 107–108). The tetramer associates through C4 symmetry instead of the 222 symmetry expected when two dimers associate together. This novel dimer-tetramer transition agrees with earlier solution studies including small angle X-ray scattering, analytical ultracentrifugation, dynamic laser light scattering and our glutaraldehyde cross-linking experiments. Comparisons with the Nef_core_—Fyn-SH3 domain complexes reveal that the PXXP motif that interacts with the SH3-domain in the dimeric form is sterically occluded in the tetramer. However the 151–180 loop that is distal to the PXXP motif and contains several protein interaction motifs remains accessible. The results suggest how changes to the oligomeric state of Nef can help it distinguish between protein partners.

## Introduction

HIV-1 Nef is an important accessory protein that is attributed with multiple distinct functions such as immune evasion [1], [2], virion infectivity [3], [4] and support for viral replication [5], [6] and survival. These functions are regulated through the interactions of Nef with more than 30 different partner proteins in the membrane anchored and cytoplasmic states respectively [7], [8], It is puzzling as to how this small ∼27 kDa protein can control multiple important functions. Vicinal to membranes, it exists as smaller oligomers such as a dimer or a trimer, as seen in the core domain (Nef_core_) complexes with the SH3 domain of Fyn Kinase (Fyn_SH3_) [9]–[11]. On the other hand small angle X-ray scattering, dynamic light scattering and analytical centrifugation studies show that the cytoplasmic form exists as a tetramer or higher oligomers that result when multiple tetramers associate together [12]. Further, a 4-fold symmetry was detected in the higher order Nef oligomers in the solution studies. The latter has been postulated to be the ‘*closed*’ form where a subset of its numerous interactions with partner proteins should be differently modulated [8]. Nef is modified co-translationally through myristoylation at the N-terminus. A population of the myristoylated form, as supported by affinity experiments, exists in the cytoplasm where the N-terminal myristoylated segment is suggestedly sequestered in the hydrophobic pockets between helices αA and αB of the C-terminal domain [12], [13].

Each Nef chain folds into two domains [14], [15]. NMR studies involving the N-terminal membrane interacting domain demonstrate that it is highly flexible and loosely structured [16]. The C-terminal core-domain functions as the oligomerization and protein interaction domain. It contains the PXXP motif (Residues 72–78) that recognizes SH3 domains of partner proteins and other interaction motifs at a 30-residue loop (Residues 149–178) distal to the PXXP motif [17]. Two groups have reported structures of the Nef_core_—Fyn_SH3_ complexes [9], [10]. The domain itself exists as a dimer in the *apo* and complexed states and represents the ‘*open*’ form of the protein. The 30-residue loop mentioned above is disordered in the complexes. This loop contains distinct motifs such as the di-Leu and EE motifs as also acidic residue clusters that interact with different sets of host/accessory proteins [8], [18]. Thus motifs at different regions of the core domain are involved in interactions with distinct subsets of protein partners for enforcing its multiple functions.

Despite earlier efforts, structural elucidation of a full-length Nef as also of the elusive ‘*closed*’ oligomeric form has not been reported so far. This has led to a gap in understanding, in molecular terms, as to how Nef modulates its interactions with partner proteins. We report the crystallization of the full-length HIV-1 Nef after experimentation with naturally occurring Nef variants from patient samples and were successful with one such sample. This Nef variant was earlier shown by our groups to exhibit pathogenicity in a *C. elegans* model [19]. The protein adopts the elusive *closed* tetrameric association in the structure. The transition from a dimer to a tetramer involves large changes to the dimeric association to form a tetramer with 4-fold symmetry. The molecular details of the dimer-tetramer transitions help explain how the protein can distinguish between different sets of protein partners when it is in the membrane-associated and cytoplasmic stages respectively. To our knowledge this is the first example where a functional dimer-tetramer transition deviates from the expected 222 symmetry to 4-fold symmetry. These large changes to the intersubunit spatial disposition involve Asp125, a highly conserved residue known to be important for viral replication and the conserved XR motif (where X is Lys or Arg) [20], [21] at the intersubunit interface. In the earlier Nef_core_—Fyn_SH3_ complexes [9], [10] the interaction of the conserved Asp was with the first residue of the motif presumably leading to the observed dimeric association, while in the present structure the interaction of the Asp shifts to the second Arg residue of the motif leading to the novel dimer-tetramer transition.

## Results & Discussion

### Crystallization and structure description

**Figure 1:**
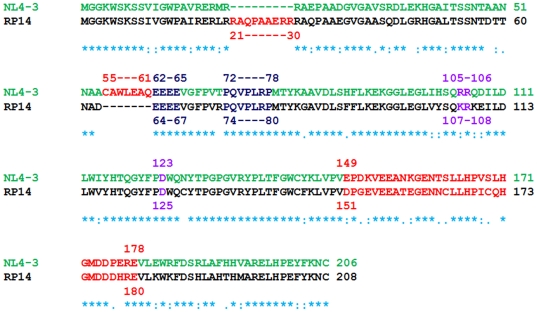
Sequence alignment of the present full-length HIV-1 Nef (NefRP14) with NL4-3. NL4-3 and RP14 sequences are shown in green and black color respectively. The main differences between the sequences involve the insertion of 9 residues after Arg20 and the deletion of the CAWLEAQ motif containing residues 55-61 in the NL4-3 sequence. The conserved Asp123 of the earlier NL4-3 sequence corresponds to Asp125 in the present structure. The XR motif involving residues 105–106 in the earlier NL4-3 sequence corresponds to residues 107–108 in the present structure. The PXXP motif in the present structure is from residues 74–80 while the 30-residue disordered loop distal to this motif ranges from residues 151–180.

We attempted crystallization of the full-length HIV-1 Nef (RP14) with several cloned samples derived from patients and were successful with the sample with sequence as deposited in the *GenBank* with accession number: GQ184340. This latter Nef variant (RP14) was also shown to be pathogenic in a *C. elegans* model [19]. Weak X-ray diffraction and crystal reproducibility necessitated extensive screening of the crystals before data collection. A comparison of the RP14 sequence with the NL4-3 strain used in the structure of the earlier reported core-domain of HIV-1 Nef is in (Figure 1). The main difference is the absence of the CAWLEAQ motif (residues 55–61) in the RP14 variant compared to the earlier NL4-3 strain and insertion of 9 amino acids after Arg20. These differences probably contribute to the successful crystallization of the present construct compared to the earlier efforts of other groups involving other Nef variants. In fact our own crystallization attempts with full-length Nef variants that contain the CAWLEAQ motif were not successful.

**Figure 2:**
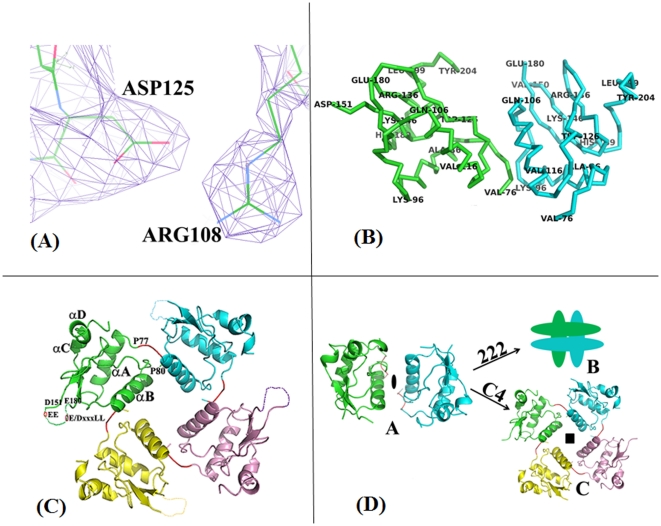
The crystal structure of HIV-1 Nef (NefRP14). (**A**) A section of the 2Fo-Fc electron density map (violet mesh) contoured at 1σ around R108 and D125. (**B**) Labeled stick representation of the two chains in the asymmetric unit. (**C**) Tetrameric association of full-length HIV-1 Nef. Individual chains are distinctly colored and shown in cartoon representation. The PXXP motif (residues 74–80) of each chain is marked in red. The α-helices of one chain are labeled for clarity. The location of protein interaction motifs in the 151–180 disordered loop is schematically indicated. (**D**) The full-length HIV-1 Nef forms a tetramer with a 4-fold symmetry (*bottom right*). This is unexpected because normally a dimer to tetramer transition involves an association that exhibits 222 symmetry as schematically illustrated in the figure. The dimer to tetramer transition in Nef involves large changes to the relative spatial disposition of the subunits of the dimer. The Nef_core_ dimeric association is shown on the *left*. The observed symmetry in the Nef tetramer crystal structure is supported by earlier small angle solution X-ray scattering experiments where tetramers and higher order oligomers with 4-fold symmetry were observed.

The asymmetric unit contains two protein chains that associate through symmetry to form a tetramer. Three lines of arguments suggest conclusively that the observed tetramer is not an artifact: firstly, earlier solution and *in vivo* studies [22] demonstrated that mutations to the corresponding Arg107 & 108 residues and Asp 125 residues in their construct severely impacted any oligomerization. Secondly, small-angle X-ray scattering experiments [12] clearly demonstrated the existence of tetramers with 4-fold symmetry and higher order oligomers that are multiples of tetramers. This is similar to our observation of 4-fold symmetry for the tetramer in the present crystal structure. Thirdly, earlier analytical centrifugation, dynamic laser light scattering and our glutaraldehyde cross-linking experiments (Figure S1) confirm the presence of the tetramer and other oligomers of Nef. The individual core-domains in the crystals are well defined in the electron density maps (Figure 2A) except for the 30-residue loop that was disordered in the earlier core domain complexes also. The domain adopts an α/β fold and contains 4 antiparallel β-strands straddled primarily by two long and two short α-helices respectively (Figure 2B, C). Details of the tertiary structure of the domain have been described by earlier workers [9], [10] and will not be mentioned here. They exhibit *r.m.s.d* values ranging between 0.69 and 0.83 Å between themselves and earlier reported structures of the domain. These values are not indicative of substantial changes to the tertiary structure as they are in the range of the average co-ordinate errors of the respective compared structures. On the other hand, the N-terminal anchor domain is disordered in the full-length structure. The presence of the full-length protein in the crystals was however confirmed by crushing a couple of crystals after washing them and running an SDS PAGE gel (Figure S2). Additionally, the crystal packing was consistent with the presence of the full-length protein. The disordered nature of the anchor domain is not surprising; as in earlier NMR studies the N-terminal domain is loosely structured and does not have a well-defined fold [18]. Additionally, in the Nef_core_—Fyn_SH3_ complex, the crystallized Nef deletion-construct consisted of residues 54–205 but the first 16 residues were disordered [9].

### HIV-1 Nef exhibits unprecedented dimer-tetramer transition

**Figure 3:**
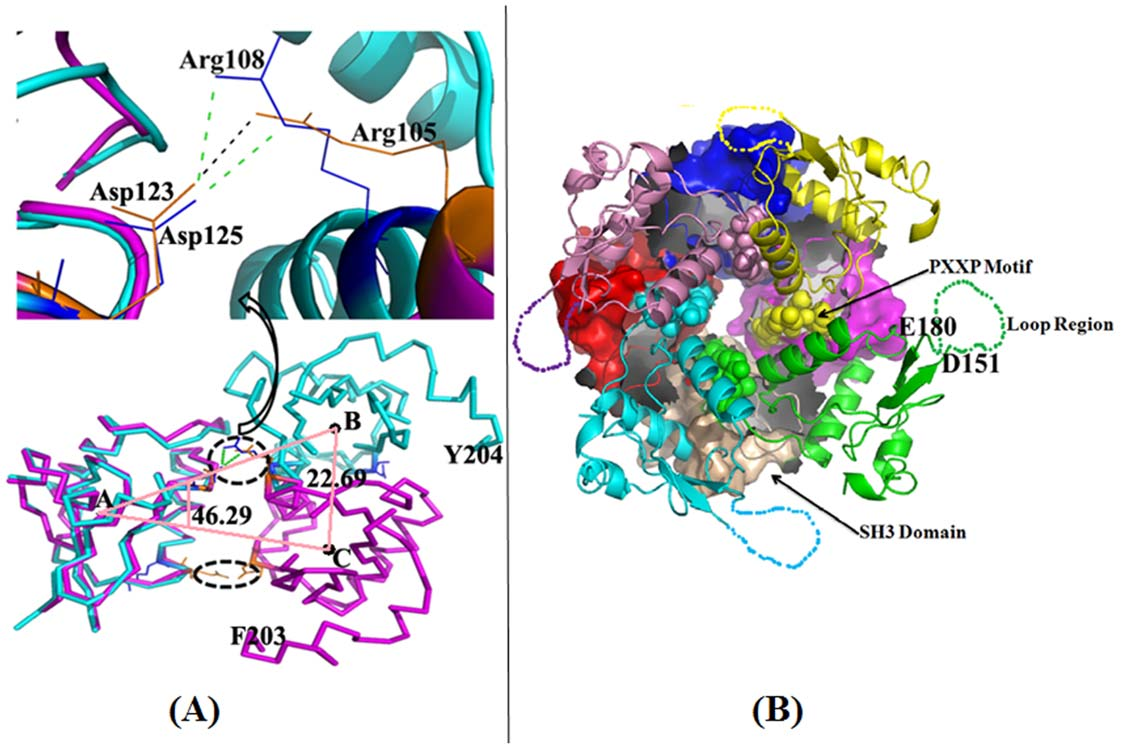
Dimer-tetramer oligomeric changes and occlusion of PXXP motif in HIV-1 Nef (NefRP14). (**A**) (*Bottom panel*) Changes to the relative spatial disposition of the two chains of the Nef_core_ dimer (*lavender*) from the earlier ‘open’ structures to that observed in the present full-length tetramer (individual chains depicted in *cyan*). One subunit from the respective associations is superposed and the relative spatial changes to the other subunit are shown. The ‘moving’ subunit is rotated by ∼174° while the center of mass is translated by ∼22.7 Å. Despite the large changes that result in the formation of the tetramer, the interactions of Asp125 with residues of the XR motif (residues 107–108) (shown as a close-up in the *upper panel*) is retained. (**b**) One chain along with the SH3 domain (surface representation) from the Nef_core_-Fyn_SH3_ complex superposed onto the tetrameric association of the full-length HIV-1Nef (cartoon representation). The SH3 domains exhibit severe steric clashes precluding their interactions with the tetrameric form of Nef. The PXXP motif (shown as spheres) cannot interact with the domain in the tetramer while the motifs present on the labeled 151–180 residue loop are exposed and can interact with other subsets of interacting protein partners.

The protein exists as a tetramer in the crystals. Surprisingly, the tetramer exhibits 4-fold symmetry as opposed to a normally expected 222 symmetry [23], [24] when two dimers associate together (Figure 2C). The observed tetrameric association is in agreement with earlier reported solution studies and small-angle X-ray scattering experiments where a tetramer and hexadecamer with 4-fold symmetry were observed [12]. In line with the earlier reports, our glutaraldehyde cross-linking experiments (Figure. S1) detected dimers, tetramers and other oligomers in the solution similar to the results of the other earlier experiments involving Nef. As per well established principles of protein oligomerization [23], [24], the tetrameric association of proteins composed of identical subunits can correspond to either C4 symmetry where the four subunits are related by a 4-fold rotation or alternatively by 222 symmetry that occurs when two dimers associate as a ‘dimer of dimers’. Deviations from these principles are rare and when present are functionally justified. The formation of the Nef tetramer involves unexpected large changes to the intersubunit disposition of the core-domains in the earlier dimer to form a tetramer with C4 symmetry (Figure 2D). Compared to the dimeric association of the earlier ‘*open*’ form, one subunit of the core-domain swings out with a relative rotation of about 174° and translation by ∼22 Å with respect to the respective center of masses (Figure 3A; bottom panel). Further association with another symmetric pair leads to the formation of the tetramer. This leads to the steric shielding of the PXXP motif in the tetramer. The functional implications of the changes that occur upon formation of the tetramer are discussed later.

### The conserved Asp125 interaction with the XR motif that is important for viral replication is retained in *closed* and *open* forms

The interactions in a dimeric interface are normally symmetrical keeping in view the two-fold symmetry of the protein-protein interaction interface. Accordingly in the Nef_core_—Fyn_SH3_ domain complex the main polar interactions occur between Asp123 and Arg105 (numbering in their construct, See Figure 1) and the reverse symmetric interactions. The Arg105 of the earlier Nef_core_ structure is the first residue of the conserved XR motif, where X is either R or K [19], [20]. In the tetrameric association the Asp125 (Asp123 of Nef_core_ structure) now interacts with the second R residue of the XR motif (Figure 3A; upper panel). A list of the interactions in the respective intersubunit interfaces of the ‘open’ and the present ‘closed’ forms is in Table 1. Earlier *in vivo* studies involving mutations to the Asp residue demonstrated that it leads to the severe deficiency to oligomerization and consequently to viral replication [25], [22]. Similarly, mutations to the XR motif also lead to severe functional consequences [22]. Very recent *in vivo* and solution studies [26] support different oligomerization modes in Nef but did identify that the XR motif and Asp125 play important roles. The present structure underscores the importance of the interaction of these two motifs in Nef oligomerization. It shows how the protein apparently can switch from a dimeric to a tetrameric association by minor changes to the intersubunit interactions. It is worth emphasizing in this context that both the residues of the XR motif are within ‘striking’ distance of the conserved Asp in both the oligomeric forms.

**Table 1:** Polar interactions less than 4 Å between the subunits in the full-length Nef tetramer.

(A) Interactions in the tetramer (Full-length Nef)		
Subunit 1	Subunit 2	Distance (Å)
Asp125-OD2	Arg108-NH2	3.1
	Arg108-NE	3.2
Leu78-O	Gly98-O	3.3

(B) Interactions in the Nef_core_ dimer		
Subunit 1	Subunit 2	Distance (Å)
Asp123-OD2	Arg105-NH2	3.3
	Arg105-NE	3.8
Arg105-NH1	Asp123-OD2	2.5
	Asp123-OD1	4.0

The interactions in the Nef_core_ dimer is also tabulated separately for ready comparison. The residue numbering follows the respective structures. An additional polar interaction in the Nef tetramer compared to the Nef_core_ dimer involves Leu78 and Gly98 respectively.

### HIV-1 Nef modulates its distinct functions through novel changes to oligomerization

Superposition of the Nef_core_—Fyn_SH3_ complex onto the tetramer shows that the binding of SH3 domains to the tetramer cannot occur as changes to the intersubunit disposition in the latter leads to severe steric conflicts (Figure 3B). The PXXP recognition motif is therefore not accessible to the SH3 domains of partner proteins in the tetramer. However accessible surface area calculations show that the accessibility of the hydrophobic pocket between the αA and αB helices is not affected when going from the ‘open’ to ‘closed’ forms. Conceptually, this will allow for the proposed sequestering of the myristoylated N-terminal segment in the hydrophobic pocket when the protein is not vicinal to membranes [12]. It is also available for its suggested role in fine-tuning interactions with other protein partners as observed in the Nef_core_—Fyn_SH3_ complexes. The 30-residue loop located distal to the PXXP motif, mentioned earlier, contains several motifs for interactions with other protein partners. An examination of the tetrameric association shows that this loop is available for interactions with protein partners (Figure 2C & 3B). An oblique support for this hypothesis is from the very recent crystal structures of the Nef –core domain, allele SF2 complexed to the SH3 domain of human Hck kinase [27]. In this latter complex the 30-residue loop is ordered and stabilized by crystal contacts with the conserved hydrophobic groove of a neighboring Nef molecule. It however does not interact with the SH3 domain of the kinase supporting distinct roles for the PXXP motif and the other protein interaction motifs located on the 30-residue loop. Thus the Nef tetramer can selectively abrogate the recognition of SH3 domains while allowing for interactions with other subsets of its repertoire of protein partners. This offers an elegant molecular mechanism for the modulation of at least a subset of the functions enforced by HIV-1 Nef in the cell. Roles of these and other postulated quaternary associations in the overall Nef functions and ‘cycle’ needs additional concerted work. However, the implications of the present are results are discussed below.

### Nef oligomers and their importance in the overall ‘Nef-cycle’

It is perplexing as to how Nef mediates the interactions with a large number of proteins, especially given its rather small size and how it results in diverse functional consequences. It is generally agreed that conformational changes based on the cellular location of Nef plays an important role. Interaction motifs are distributed in the protein in primarily two regions; the first involves the PXXP motif and the other involves motifs situated in the 30-residue loop. The oligomeric changes exhibited by Nef were associated with the enforcing of the multiple distinct functions of the protein [8], [18]. It is also known that Nef exists as smaller oligomers in the vicinity of membranes where it interacts with a distinct set of proteins while it interacts with other proteins in the cytoplasm [8], [18]. The myristoylated N-terminus that interacts with membranes has to be sequestered in the cytoplasmic stage. The earlier Nef_core_—Fyn_SH3_ complex, where the association is dimeric, gave a detailed picture, perhaps reminiscent of the interactions of Nef in the vicinity of membranes. Other groups have reported detailed solution studies involving the cytoplasmic form of Nef including solution X-ray scattering, analytical ultracentrifugation and dynamic laser light scattering [12]. These studies have revealed that Nef exists as a tetramer with 4-fold symmetry and also that it forms a hexadecamer when 4 such tetramers associate together. These earlier studies supported that large changes should take place in the Nef oligomeric association when going from the membrane-associated ‘open’ form to the cytoplasmic ‘closed’ form. The relationship between this higher oligomeric form and function however remained unclear. The present work affords definitive insights as to how the oligomeric changes in Nef enable it to distinguish between subsets of its multiple interacting partners. The shielding of the PXXP motif of Nef in the cytoplasmic stage prevents the binding of SH3 domains of selected proteins that presumably interact with it in the lower order membrane-associated form. It however allows for Nef to interact with other selected partners in the cytoplasmic stage since the 151–180 loop containing other interaction motifs is accessible. The structure also affords insights as to how the myristoylated N-terminus of Nef that is membrane-associated in the ‘open’ form, and also binds to the core domain of Nef [13], can be occluded in the cytoplasmic stage since the earlier suggested [12] binding pocket between helices αA and αB (Figure 2C) remains accessible in the tetramer. Consistent with these suggestions a recent report involving [27] the Nef_SF2_ –Hck_SH3-B6_ complex and associated Isothermal calorimetry studies showed that myristoylation of Nef does not interfere with the SH3 domain binding activity. It also showed that the 151–180 loop does not interact with the predicted binding site of myristoylated peptides in the hydrophobic groove between helices αA and αB. Future complexes of Nef with proteins that interact with motifs in the 151–180 loop should reveal molecular details of interactions with the latter. These complexes do not preclude additional relative displacements between the subunits that might occur on interactions with the target proteins. A support for this proposal comes from very recent structural studies involving the core domain of SIVmac239 isolate of Nef (SIVmac239 Nef_core_) with two interacting peptides derived from the signalling ζ subunit of the T-cell receptor (TCRζ) [28]. In these latter studies a relative 10° rotation between the respective core domains was observed when comparing the two complexes. Multiple modes of oligomerization in the SIV ortholog of Nef were also shown recently through solution and cell based studies [26]. In these studies alternate models of dimer formation in SIV-Nef was suggested. The authors further reported that the di-basic XR motif is important for these alternate modes of oligomerization also. The present work involving the HIV-1 Nef represents novel insights to aspects of the large quaternary structural and other changes that are apparently exploited by Nef to enforce its functions.

### Conclusions

Overall, the present work shows that there is nothing ‘*closed*’ about the structure of the cytoplasmic form of Nef and that the protein controls interactions with different partners in novel ways. The shielding of the PXXP motif that interacts with the SH3 domains in the tetramer is unanticipated. We know of no other example where a dimer breaks its 2-fold symmetry to form a 4-fold symmetric tetramer. The unprecedented changes however retain the key interactions of Asp125 with the XR motif that were earlier shown to be essential for viral replication [22]. Furthermore the importance of the Asp125- XR motif interactions in oligomeric assembly is underscored by the present work. The nature of the bond (salt-bridge) apparently allows for the subunits to be associated with each other while undergoing the large relative displacements when going from the dimeric to tetrameric forms. Given the highly conserved and essential nature of the Asp125- XR interactions it remains to be seen whether these can be disrupted as an innovative route to the development of novel anti HIV therapeutics.

## Materials and Methods

### Ethics statement

The study was approved by the Human Ethical Committee of the ‘Chhatrapati Shahuji Maharaj Medical University (old name: King George Medical University), Lucknow-226003 (UP) India, with Ref. Code: XVIII ECM/P9. The written informed consent was obtained from all study participants.

### Genomic DNA isolation, PCR amplification and sequencing of HIV-1 Nef gene

Peripheral Blood Mononuclear Cells (PBMCs) were isolated from a random HIV-1 infected individual, after informed consent, using the standard Ficoll density gradient centrifugation method (Sigma). The study was approved by the Human Ethical Committee of the ‘Chhatrapati Shahuji Maharaj Medical University’ (old name: King George Medical University), Lucknow-226003 (UP) India, with Ref. Code: XVIII ECM/P9. The written informed consent was obtained from all study participants. Genomic DNA was isolated from fresh PBMCs by a genomic extraction kit (Invitrogen). Nested PCR approach [29] was used to amplify the Nef gene using different sets of primers, outer forward primers POLU 5′-CCCTAYAACCMCARAGYCARGG-3′ and outer reverse LTRR 5′-GACTACGGCCGTCTGAGGGATCTCTAGYTACCA-3′ and Inner forward Primer NOFP 5′-ATACCTASAMGAATMAGACACARGG-3′ and inner reverse primer NORP 5′-CTGCTTATATGCAGCATCTGAGGG-3′) [30]. The amplified PCR fragments were cloned into the pDrive T/A cloning vector (Invitrogen). Three clones per sample were sequenced using M13 forward and reverse sequencing primers using the Bigdye terminator chemistry on an automated sequencer (ABI prism 310 genetic analyzer, Applied Biosystems). The sequences obtained were assembled using the AutoAssembler (ABI PRISM) DNA sequence assembly software (Figure 1). The sequence of the NefRP14 gene was submitted to the GenBank with the accession number: GQ184340.

### Cloning, Over-expression and Purification of His-tagged HIV-1 Nef

The Nef gene was amplified and cloned into the TA cloning vector (Invitrogen) using forward primer P14BF 5′-CATGGATCCCAGCAGCAGAAAGGAGA-3′ and reverse primer NX1R CCAGGTCTAGACCCAGCGGAAAGTCCC-3′ with BamHI restriction sites at their overhangs and subsequently subcloned into the pET28a bacterial expression vector (Novagen). The pET-28a-Nef plasmid containing hexahistidine tags was transformed into the C41 bacterial strain. The single colony culture was induced by the addition of 0.8 mM IPTG at 37°C for 6 hrs. Before purification the expression and solubility was checked on SDS-PAGE. The protein was purified by affinity chromatography using a Ni-NTA column and Tris-Cl (25 mM pH 8.8), NaCl (50 mM) buffer. The protein eluted at 350 mM imidazole concentration and was subjected to 12% SDS PAGE analysis where the Page Ruler™ Prestained Protein Ladder (Fermentas Cat. SM0671) was used as a molecular weight marker (Figure S2). Subsequently gel filtration experiments were carried out using a Superdex-200 HR 10/30 column mounted on an AKTA FPLC (GE Healthcare). The column was equilibrated with 50 mM Tris-HCl (pH 8.5), 100 mM NaCl, 5 mM EDTA and 2 mM 2-mercaptoethanol. The purified protein was concentrated using a 10 kDa cutoff centricon (Ms/Amicon) for further use.

### Glutaraldehyde cross-linking

The purified Nef protein was incubated with increasing concentrations of Glutaraldehyde (0.0%, 0.025%, 0.05%, 0.075% and 0.1%) on ice for 15 minutes. Respective samples were prepared and run on 12% SDS-PAGE. The gel was visualized after silver staining it. The gel was subjected to western blotting using anti-His antibody to unambiguously identify the oligomeric bands (Figure S1).

### Crystallization, data collection and processing

Crystallization trials using the hanging drop method (2∶1 ratio of protein and precipitant solutions) were carried out using the purified Nef at a concentration of ∼3.7 mg/ml. Various constructs based on the Nef variants from patient samples were used for crystallization trials and ultimately we were successful with one such construct for reasons outlined in ‘Results’. The crystals of Nef (GenBank accession number: GQ184340) was obtained in the condition with 0.1 M Tris (pH 8.5), 0.2 M Ammonium sulfate, 20% PEG 8000. The full length HIV-1 Nef formed hexagonal shaped crystals. The presence of the full-length protein in the crystals was confirmed by crushing a couple of crystals after washing them and running an SDS PAGE gel. Crystal diffraction and poor reproducibility were problems faced during the experiments and in hindsight were probably due to the disordered N-terminal domain as explained in ‘Results’. The crystals were flash frozen after briefly soaking them in the precipitant solution supplemented with 30% glycerol for data collection. A Rigaku Micromax 007HF X-ray generator was coupled to a MAR345-DTB detector with VARIMAX-HF optics and used in the data collection. An Oxford Cryostream700 instrument was used to maintain the temperature at 100°K. Most of the crystals diffract rather weakly and therefore many crystals were screened before the data collection. The crystals belong to space group P6_3_22 (a = b = 123.03; c = 130.4 Å) and diffract to 3.5 Å. The data collection and refinement statistics are summarized in Table 2. Data integration, reduction and scaling were performed using MOSFLM [31] and its companion program SCALA [32]. Data were overall complete to 94.8% with an average redundancy of 5.7. Crystal mosaicity values refined to 0.67. The Matthews's coefficient of 2.37 Å^3^/Da or solvent content of about 48.7% suggested that 2 molecules were there in the asymmetric unit.

**Table 2:** Data collection and refinement statistics for full length HIV-1 Nef.

	HIV-1 Nef
**Data collection**	
Space group	P6_3_22
Cell dimensions	
*a*, *b*, *c* (Å)	123.03, 123.03, 130.4
α,β,γ (°)	90.0, 90.0, 120.0
Resolution (Å)	3.5 (3.72-3.5)*
*R*_merge_	0.12(0.67)
*I*/σ*I*	7.1(3.9)
Completeness (%)	94.8(89.6)
Redundancy	5.7(4.5)
**Refinement**	
Resolution (Å)	29.6-3.5
No. reflections	7262
*R*_work_/*R*_free_	22.46/28.80
No. atoms	
Protein	1686
Ligand/ion	-
Water	49
*B*-factors	
Protein	37
Ligand/ion	-
Water	30
R.m.s. deviations	
Bond lengths (Å)	0.01
Bond angles (°)	1.4

Data were collected using 1 flash frozen crystal at 100°K.

* Values in parentheses are for highest-resolution shell.

### Structure solution, refinement, model building and validation

The structure was solved using molecular replacement (MR) techniques employing the program Phaser [33]. Since models are available only of the C- and N-terminal domains of the protein respectively, we followed a strategy of independently searching for the two domains. Three structures of the core domain were superposed using PROFIT (http://www.bioinf.org.uk/software/profit) and parts of 1AVV, which structurally align well, were identified visually. Non-conserved residues were converted to alanine in the search model. Data in the resolution range 29.6-3.5 Å were used for molecular replacement calculation. Calculations were carried out in all the sub-groups of P622. The correct solution was in the space group P6_3_22 and the packing was examined carefully. There are two independent chains in the asymmetric unit. There was also enough space to accommodate the N-terminal domain that was yet to be placed. Calculations involving the available myristoylated and non-myristoylated structures of the N-terminal domain were not successful despite various parameters like the resolution range *etc* being changed. An examination of the electron density maps at this stage suggested that the N-terminal domains might be disordered as suggested by weak trailing density at the start of the C-terminal core domain. The core domain itself was well defined in the electron density maps. We therefore started model building with a view that improved phases after model building might result in better maps for modeling the N-terminal domain. Ultimately we realized that the N-terminal domain is disordered, although it is present in the crystals (Figure S2). A long loop of the core domain from 151–180 is also disordered in the crystal structure. This is disordered in the earlier reported structures of the core-domain also. Refinements were carried out using REFMAC5 [34] in the initial stages while model building was carried out using TURBO-FRODO [35] and COOT. In the later stages we took recourse to the PHENIX package [36] for the refinements. The starting models derived from the molecular replacement calculations were subjected to rigid body refinement with the respective subunits treated as rigid units. An independent set consisting of 5% of the diffraction data were set aside to monitor the R-free value during the course of refinements. NCS constraints were imposed till the penultimate cycles of refinement. The crystallographic R-factor and R-free [37] were monitored at each stage and omit maps were checked to avoid model bias. These values stand at 22.46 and 28.8% respectively in the final model. It is possible that the disorder of the large N-terminal domain could have contributed to relatively weaker diffraction of the crystals. PROCHECK was used to assess the quality of the model after every round of refinement.

### Accession numbers

The coordinates and structure factors have been deposited in the Protein Data Bank (http://www.rcsb.org) with accession codes 2XI1 and R2XI1SF respectively.

## Supporting Information

Figure S1 **Glutaraldehyde crosslinking experiments with HIV-1Nef.** (**A**)The Nef protein was crosslinked by glutaraldehyde with 0.0% (control), 0.025%, 0.05%, 0.075%, 0.1% concentrations respectively and subsequently run on 12% SDS-PAGE. The gel was silver stained to visualize the different oligomeric forms of Nef protein. (**B**) Since the purified protein contains a hexa-His tag, we unambiguously identified the oligomeric forms by western blotting with anti-His antibody.
(PDF)

Figure S2 **SDS-PAGE gel of crushed HIV-1 Nef crystals (** ***right*** **) run along with the molecular weight marker from** ***Fermentas*** **.** A sample gel from a purification run is shown on the *left* for comparison. Lane L5 corresponds to the purified eluted protein from the Ni-NTA column while lanes L1-L4 correspond to the other stages of purification including the wash cycles.
(PDF)
