# Supplementary material for: A Novel Dimer-Tetramer Transition Captured by the Crystal Structure of the HIV-1 Nef
Source: PLoS One. 2011 Nov 2;6(11):e26629. doi: 10.1371/journal.pone.0026629 (PMC3206816; doi:10.1371/journal.pone.0026629)

Supplementary Figure 1

Glutaraldehyde crosslinking experiments with HIV-1Nef:
(**A**)The Nef protein was crosslinked by glutaraldehyde with 0.0% (control), 0.025%, 0.05%, 0.075%, 0.1% concentrations respectively and subsequently run on 12% SDS-PAGE. The gel was silver stained to visualize the different oligomeric forms of Nef protein.
(B) Since the purified protein contains a hexa-His tag, we unambiguously identified the oligomeric forms by western blotting with anti-His antibody.

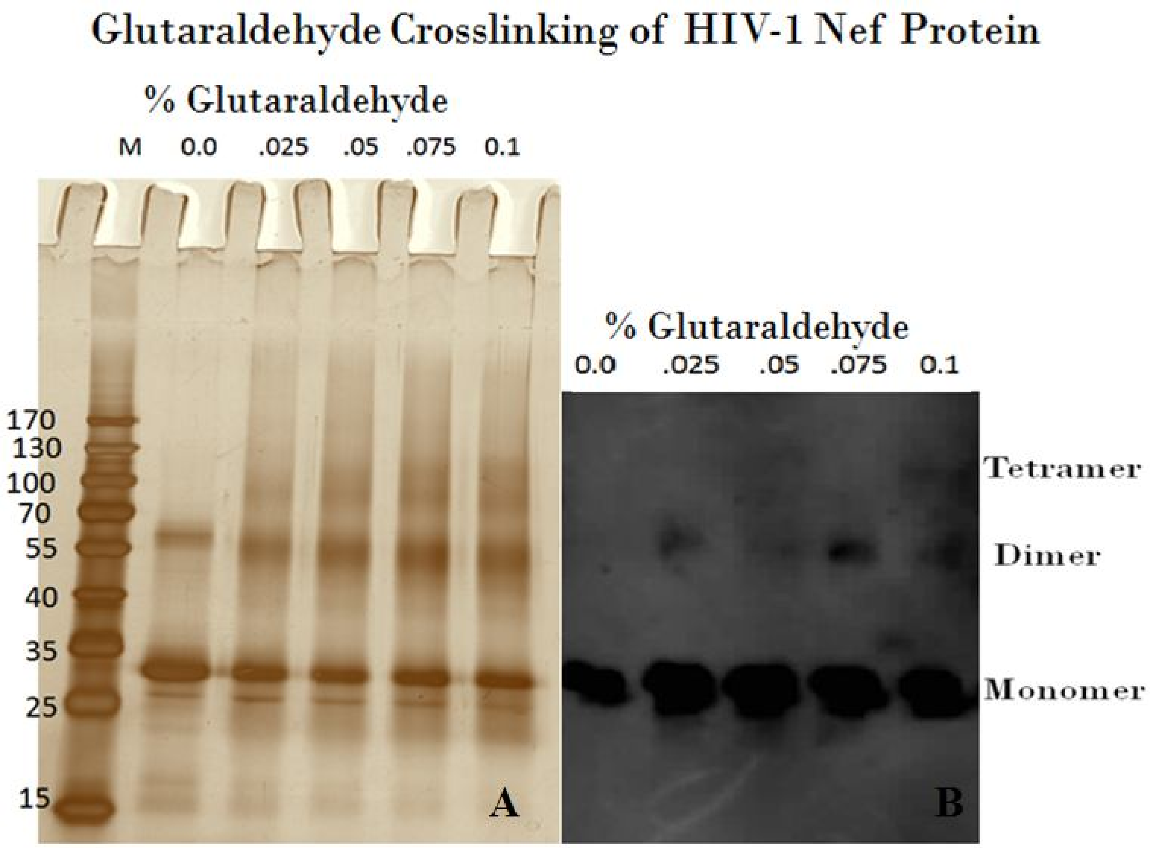

Supplement: Figure S1 — Glutaraldehyde crosslinking experiments with HIV-1Nef. (A)The Nef protein was crosslinked by glutaraldehyde with 0.0% (control), 0.025%, 0.05%, 0.075%, 0.1% concentrations respectively and subsequently run on 12% SDS-PAGE. The gel was silver stained to visualize the different oligomeric forms of Nef protein. (B) Since the purified protein contains a hexa-His tag, we unambiguously identified the oligomeric forms by western blotting with anti-His antibody. (PDF) [file pone.0026629.s001.pdf]
