# Supplementary material for: A Novel Dimer-Tetramer Transition Captured by the Crystal Structure of the HIV-1 Nef
Source: PLoS One. 2011 Nov 2;6(11):e26629. doi: 10.1371/journal.pone.0026629 (PMC3206816; doi:10.1371/journal.pone.0026629)

Supplementary Figure 2

SDS-PAGE gel of crushed HIV-1 Nef crystals (*right*) run along with the molecular weight marker from *Fermentas*. A sample gel from a purification run is shown on the *left* for comparison. Lane L5 corresponds to the purified eluted protein from the Ni-NTA column while lanes L1-L4 correspond to the other stages of purification including the wash cycles.

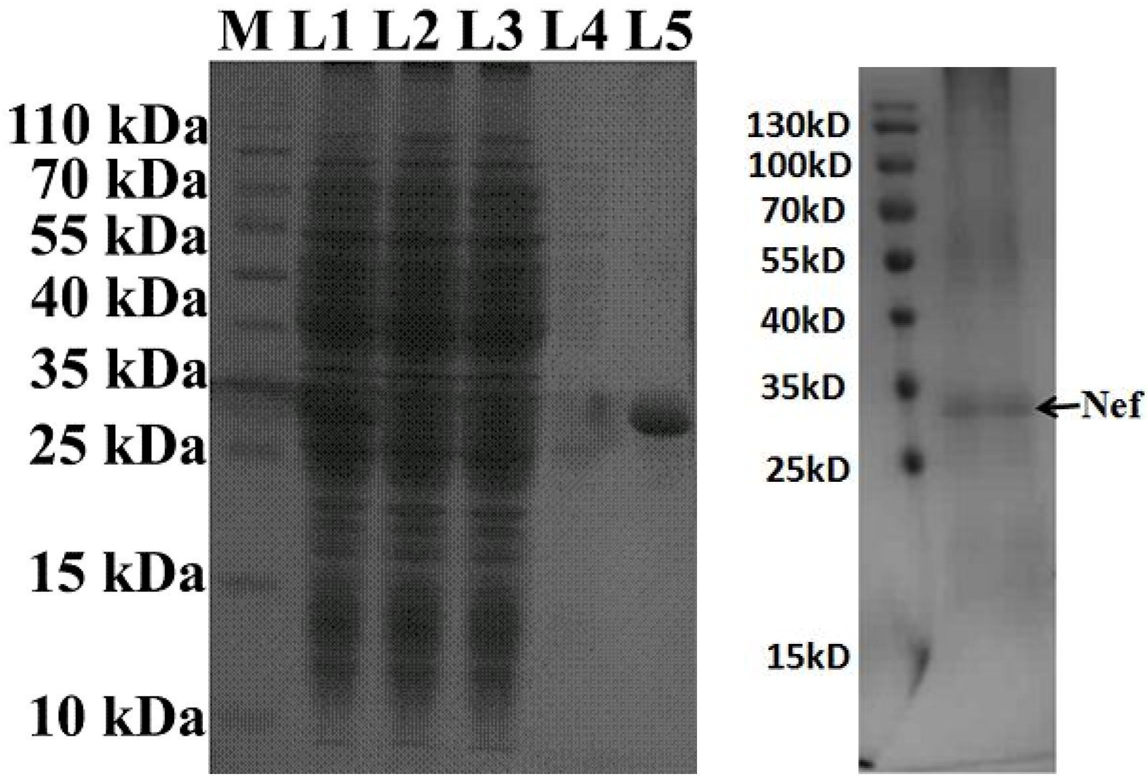

Supplement: Figure S2 — SDS-PAGE gel of crushed HIV-1 Nef crystals ( right ) run along with the molecular weight marker from Fermentas . A sample gel from a purification run is shown on the left for comparison. Lane L5 corresponds to the purified eluted protein from the Ni-NTA column while lanes L1-L4 correspond to the other stages of purification including the wash cycles. (PDF) [file pone.0026629.s002.pdf]
